# Natural Splice Variant of MHC Class I Cytoplasmic Tail Enhances Dendritic Cell-Induced CD8^+^ T-Cell Responses and Boosts Anti-Tumor Immunity

**DOI:** 10.1371/journal.pone.0022939

**Published:** 2011-08-10

**Authors:** Tania G. Rodríguez-Cruz, Shujuan Liu, Jahan S. Khalili, Mayra Whittington, Minying Zhang, Willem Overwijk, Gregory Lizée

**Affiliations:** Departments of Melanoma Medical Oncology and Immunology, University of Texas M. D. Anderson Cancer Center, Houston, Texas, United States of America; Institut national de la santé et de la recherche médicale (INSERM), France

## Abstract

Dendritic cell (DC)-mediated presentation of MHC class I (MHC-I)/peptide complexes is a crucial first step in the priming of CTL responses, and the cytoplasmic tail of MHC-I plays an important role in modulating this process. Several species express a splice variant of the MHC-I tail that deletes exon 7-encoding amino acids (Δ7), including a conserved serine phosphorylation site. Previously, it has been shown that Δ7 MHC-I molecules demonstrate extended DC surface half-lives, and that mice expressing Δ7-K^b^ generate significantly augmented CTL responses to viral challenge. Herein, we show that Δ7-D^b^-expressing DCs stimulated significantly more proliferation and much higher cytokine secretion by melanoma antigen-specific (Pmel-1) T cells. Moreover, in combination with adoptive Pmel-1 T-cell transfer, Δ7-D^b^ DCs were superior to WT-D^b^ DCs at stimulating anti-tumor responses against established B16 melanoma tumors, significantly extending mouse survival. Human DCs engineered to express Δ7-HLA-A*0201 showed similarly enhanced CTL stimulatory capacity. Further studies demonstrated impaired lateral membrane movement and clustering of human Δ7-MHC-I/peptide complexes, resulting in significantly increased bioavailability of MHC-I/peptide complexes for specific CD8^+^ T cells. Collectively, these data suggest that targeting exon 7-encoded MHC-I cytoplasmic determinants in DC vaccines has the potential to increase CD8^+^ T-cell stimulatory capacity and substantially improve their clinical efficacy.

## Introduction

Presentation of MHC class I (MHC-I)/peptide complexes on the cell surface of antigen presenting cells (APC) is crucial first step in the activation of cytotoxic T lymphocyte (CTL)-mediated anti-viral and anti-tumor immune responses [1]. Amongst APCs, activated DCs are by far the most potent for initiating CD8^+^ T-cell responses; thus, they have held great promise for use in vaccines aimed at eliciting or boosting pathogen- or tumor antigen-specific CTLs [2], [3]. Many studies have reported strong induction of CTL responses following DC-based vaccination in both animal models and in selected clinical trials involving cancer patients and those harboring chronic viral infections [4]–[8]. However, although the majority of experimental DC vaccines have been successful at generating antigen-specific CTLs, clinical responses have remained sporadic, underscoring a need to improve the efficacy of DC-based vaccination [9]–[11].

A number of studies have demonstrated that the ∼35 amino acid cytoplasmic tail of MHC-I plays a critical role in intracellular trafficking, DC-mediated antigen presentation and CTL priming [12]–[15]. Encoded by two separate exons (6 and 7) and containing a number of highly conserved features, it has been shown that deletion of the entire MHC-I cytoplasmic tail results in a complete abrogation of anti-viral CTL responses *in vivo* [13]. Subsequently, it was shown that a membrane-proximal tyrosine residue (Tyr-320) encoded by exon 6 forms part of a putative endocytic motif which is required for proper MHC-I trafficking through DC endosomal compartments, cross-presentation of exogenous antigens, and anti-viral CTL priming [14], [15]. By contrast, deletion of thirteen exon 7-encoded cytoplasmic amino acids led to a significant enhancement of anti-viral CTL responses *in vivo* [14]. This is of particular interest because exon 7-deleted MHC-I (Δ7) is a natural splice variant expressed by a number of diverse species, and this isoform lacks at least one conserved serine phosphorylation site, Ser-335 [16]–[20]. Exon 7-deleted MHC-I molecules have been shown to exhibit delayed internalization in a number of cell types, including DCs [12], [15], [21]. However, unlike Tyr-320-mutated MHC-I, Δ7 molecules retain their ability to recycle through DC endosomal compartments, and appear to be fully functional at acquiring and presenting both endogenous and exogenous antigens [15], [22].

In DCs, wild-type (WT) surface MHC-I molecules are rapidly and constitutively internalized and recycled through endocytic compartments [15], [23]–[25]. This cytoplasmic tail-dependent process limits the surface half-life of MHC-I/peptide complexes and, of critical importance for antigen-loaded DC-based vaccines, likely places severe constraints on the availability of these ligands for recognition by cognate CD8^+^ T cells. We reasoned that expression of the Δ7 MHC-I isoform, with its combined attributes of impaired cell surface internalization plus intact cross-presentation capacity, might facilitate superior antigen presentation and therefore more efficient CTL activation. To test this hypothesis, we compared the relative capacity of WT and Δ7 MHC-I molecules to stimulate antigen-specific CTL responses in the context a DC-based vaccine. We report here that exon 7-deleted MHC-I molecules stimulated superior viral-specific and tumor-specific CTL responses in both murine and human systems, and provided a significant therapeutic advantage in a tumor treatment model of B16 melanoma. Collectively, these results provide a general strategy for augmenting MHC-I-mediated antigen presentation, and suggest that direct targeting of exon 7-encoded cytoplasmic determinants in human DC vaccines may substantially improve their capacity to stimulate CTL-mediated immunity.

## Results

**Figure 1:**
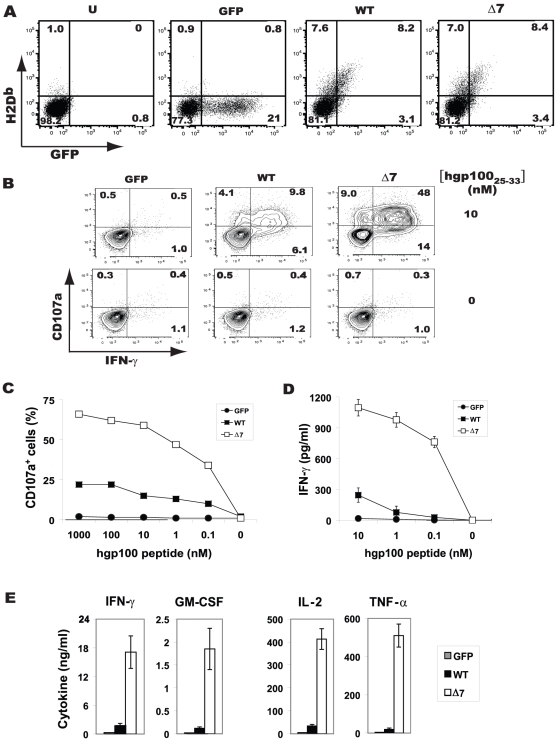
DC-mediated antigen presentation by exon 7-deleted H-2D^b^ significantly augments CD8^+^ T-cell effector function. (**A**) Bone marrow-derived DCs were transduced to express either wild-type (WT) H-2D^b^/GFP, exon 7-deleted (Δ7) H-2D^b^/GFP, or GFP alone at comparable levels, as determined by flow cytometry. (**B to D**) Transduced DCs were pulsed with the indicated concentrations of hgp100(25–33) peptide, washed, and co-cultured with gp100-specific Pmel-1 effector CD8^+^ T cells, followed by (**B**) flow cytometric analysis after 4 h to determine intracellular IFN-γ production and (**C**) CD107a expression. (**D**) IFN-γ in 18 h co-culture supernatants, as determined by ELISA. (**E**) Cytokine release by naïve Pmel-1 T cells stimulated with transduced, hgp100 peptide-pulsed (10 nM) DCs, as determined by Luminex after 72 h co-culture. All results are representative of a minimum of 4 replicate experiments. U, untransduced DCs.

In order to directly assess the CD8^+^ T-cell stimulatory capacity of exon 7-deleted MHC-I, we utilized lentiviral vector-mediated gene delivery to express comparable amounts of WT H-2D^b^ or Δ7 H-2D^b^ in bone marrow-derived DCs derived from DBA/2 mice (haplotype H-2^d^). Green fluorescent protein (GFP) was also expressed from a downstream internal ribosome entry site (IRES), and an empty-IRES-GFP vector was used as a negative control (Fig. S1A). DC transduction efficiencies typically ranged from ∼15 to 30%, as assessed by flow cytometry (Fig. 1A). Transduced DCs were pulsed with titrated amounts of a D^b^-restricted melanoma tumor-associated antigen hgp100, and used to stimulate gp100-specific Pmel-1 CD8^+^ effector T- cells *in vitro*. After 4 h of co-culture, T cells were assessed by flow cytometry for both surface expression of CD107a (a marker of degranulation) and intracellular IFN-γ. As shown in Fig. 1B, Δ7-D^b^ transduced DCs stimulated a much higher percentage of Pmel-1 T-cells compared to DCs expressing WT-D^b^ (71% vs. 20%). Similar results were observed for stimulation of degranulation and cytokine production across a range of peptide concentrations (Figs. 1C and 1D). Notably, Δ7-D^b^-expressing DCs were capable of inducing IFN-γ production at peptide concentrations at least 10-fold lower than the lower limit of cytokine detection for DCs expressing WT-D^b^ (Fig. 1D). Moreover, in the context of an *in vitro* priming assay, Δ7-D^b^ DCs elicited markedly higher levels of inflammatory cytokines IFN-γ, IL-2, TNF-α, and GM-CSF from stimulated naïve Pmel-1 T-cells (Fig. 1E).

**Figure 2:**
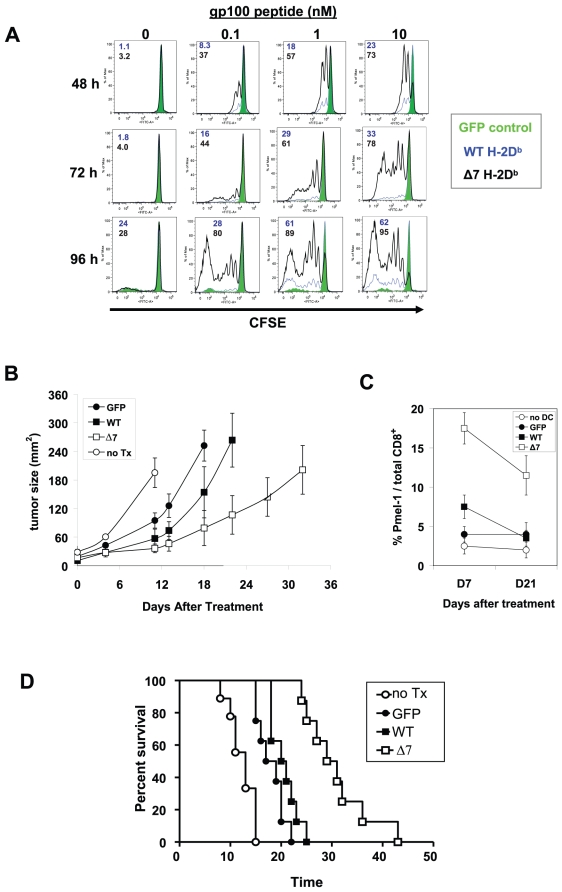
Exon 7-deleted H-2D^b^ induces enhanced CD8^+^ T-cell proliferation and improved anti-tumor responses. (**A**) WT-H-2D^b^/GFP-, Δ7-H-2D^b^/GFP-, or GFP-tranduced DCs were pulsed with titrated concentrations of hgp100 (25–33) peptide, washed, and used to stimulate CFSE-labeled, naïve Pmel-1 CD8^+^ T cells. Flow cytometric analysis of gated Pmel-1 cells indicates extent of T-cell proliferation at d2, d3, and d4 of co-culture, as measured by CFSE dilution. Numbers on histograms represent the percentage of divided T cells following stimulation with DCs expressing WT-H2D^b^ (blue) or Δ7-H2D^b^ (black). (**B to D**) Mice bearing established, subcutaneous B16 tumors were treated by co-adoptive transfer of Pmel-1 CD8^+^ T cells and transduced DCs pulsed with hgp100 peptide (300 nM), followed by 3 days of IL-2. (**B**) B16 tumor growth, as monitored following adoptive transfer. (**C**) Relative percentages of Pmel-1 T-cells in the peripheral blood of treated animals, as determined by flow cytometry at d7 and d21 post-treatment. (**D**) Kaplan-Meier survival analysis of treated mice. *N = 8* mice per group. All data are representative of a minimum of 3 replicate experiments.

We next assessed whether Δ7-D^b^ DCs could also stimulate enhanced naïve CD8^+^ T-cell proliferation. Naïve Pmel-1 T cells were labeled with CFSE and co-cultured for 4 days with either WT-D^b^ or Δ7-D^b^-expressing DCs pulsed with titrated doses of hgp100. As shown in Fig. 2A, DCs expressing Δ7-D^b^ stimulated significantly higher levels of Pmel-1 T-cell proliferation under all conditions tested. The most striking differences were observed at the lowest peptide concentrations: WT-D^b^-expressing DCs pulsed with 0.1 nM hgp100 peptide stimulated negligible Pmel-1 T-cell proliferation by day 4, whereas Δ7-D^b^ DCs pulsed with the same dose of antigen stimulated robust proliferation (Fig. S1B). In order to compare DC vaccine-induced T-cell proliferation *in vivo*, peptide-pulsed DCs and naïve Pmel-1 T cells were co-injected i.v. into C57BL/6 recipient mice. Following injection, peripheral blood was analyzed at different time points to quantitate Pmel-1 T cells. Similar to the *in vitro* results, mice injected with Δ7-D^b^ DCs had peripheral blood Pmel-1 T-cell percentages at day 6 that were ∼4-fold higher than mice injected with WT-D^b^ DCs (16% vs. 4%, Fig. S1C).

We next investigated whether Δ7 MHC-I-expressing DCs could induce better antigen-specific anti-tumor responses *in vivo* using a well-established adoptive Pmel-1 T-cell transfer model to treat gp100-expressing B16 melanoma tumors [6], [26]. Under this treatment protocol, mice with 7-day subcutaneous B16 tumors were treated by i.v. co-administration of Pmel-1 T cells and hgp100 peptide-pulsed DCs. As shown in Fig. 2B, mice treated with a Δ7-D^b^ DC vaccine demonstrated significantly delayed tumor growth compared to mice administered a WT-D^b^ DC vaccine (*p = 0.04* at day 23). This also correlated well with the percentage of Pmel-1 T cells in the blood of tumor-bearing animals, which was higher in the Δ7-D^b^ DC group at days 7 and 21 following adoptive transfer (Fig. 2C). Importantly, mice treated with Δ7-D^b^ DCs showed a significant survival benefit compared to mice treated with WT-D^b^ DCs, surviving an average of 50% longer following treatment (mean survival 31 d vs. 20 d, *p = 0.0004*, Fig. 2D). Collectively, these results demonstrated that Δ7 MHC-I could provide a substantial advantage over WT MHC-I for stimulating anti-tumor CTL responses in a DC-based cancer vaccine setting.

**Figure 3:**
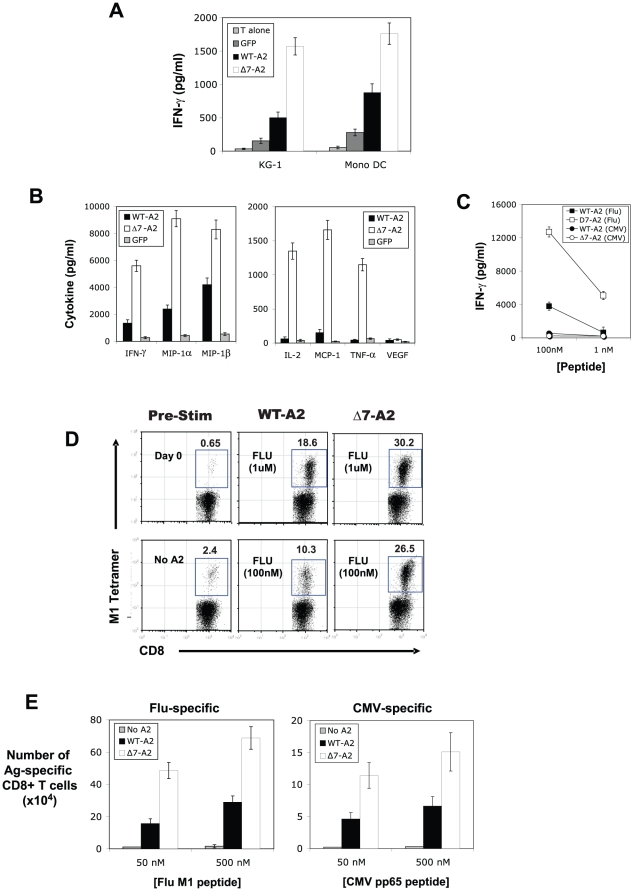
Exon 7-deleted HLA-A*0201 augments human CD8^+^ T-cell inflammatory cytokine secretion and *in vitro* proliferation. Human KG-1 cells and primary CD34^+^ DCs were transduced to express either wild-type HLA-A*0201 (WT-A2) or Δ7-HLA-A*0201 (Δ7-A2). (**A**) Transduced DCs were pulsed with MART-1 peptide (100 nM), washed, and co-cultured with MART-1-specific CD8^+^ T cells. Antigen-specific IFN-γ secretion was then determined by ELISA of overnight culture supernatants. (**B to D**) Transduced KG-1 cells were pulsed with FluM1 peptide (100 nM), and used to stimulate influenza-specific CD8^+^ T cells isolated from normal donors. (**B**) Specific inflammatory cytokine release in response to DC stimulation for 18 h, as determined by Luminex. (**C**) Specific IFN-γ secretion in response to titrated amounts of FluM1 peptide. (**D**) *In vitro* expansion of FluM1-specific CD8^+^ T cells following 8d stimulation with FluM1-pulsed KG-1 cells, as determined by tetramer analysis. (**E**) Comparison of the total number of FluM1 and CMVpp65-specific CD8^+^ T cells following a single *in vitro* stimulation with peptide-pulsed WT-A2 or Δ7-A2-expressing KG-1 cells. All data are representative of at least 4 replicate experiments.

The results from the murine studies prompted us to next assess whether the Δ7 isoform of HLA class I could similarly boost human CTL responses. Thus, we designed lentiviral vectors to express both Δ7 and WT isoforms of HLA-A*0201, and used them to transduce both CD34^+^-derived DCs and the human KG-1 DC-like cell line [27] to express comparable surface levels of HLA-A*0201 (Figs. S2A and B). Transduced DCs were then pulsed with the HLA-A*0201-restricted melanoma tumor-associated antigen MART-1 and used to stimulate MART-1-specific CD8^+^ tumor-infiltrating lymphocytes (TIL). Similar to the murine studies, Δ7-A2 DCs were superior to WT-A2 DCs at eliciting MART-1-specific IFN-γ production from TIL, whether expressed by KG-1 cells or transduced primary CD34^+^ DCs (Fig. 3A). We also compared the relative abilities of peptide-pulsed DCs to stimulate influenza matrix protein 1 (Flu M1)-specific CD8^+^ T cells derived from the peripheral blood mononuclear cells (PBMC) of normal donors. As shown in Fig. 3B, KG-1 cells expressing Δ7-A2 elicited significantly greater Flu M1-specific inflammatory cytokine/chemokine production compared to WT-A2 KG-1 cells, with IFN-γ, MIP-1α, and MIP-1β secretion being 3- to 5-fold higher. In addition, IL-2, MCP-1, and TNF-α levels were typically >10-fold higher than WT-A2 stimulated T-cell cultures, which often had barely detectable levels of these cytokines. As observed previously, Δ7-A2 DCs were capable of stimulating Flu M1-specific IFN-γ secretion at peptide doses >10-fold lower than the minimal threshold observed for WT-A2 KG-1 cells (Fig. 3C).

Transduced human DCs were also assessed for their capacity to stimulate the *in vitro* expansion of HLA-A*0201-restricted CD8^+^ T-cells from normal donor PBMC. As shown in Fig. 3D, Flu M1 peptide-pulsed Δ7-A2 DCs consistently induced superior expansion of Flu M1-specific T-cells compared with DCs expressing WT-A2, an effect that was most pronounced at limiting peptide concentrations. Similar results were also observed for the expansion of CMV pp65-specific T cells from PBMC following a single round of *in vitro* stimulation with transduced DCs (Fig. 3E). Despite their distinct proliferation indices, CD8^+^ T cells expanded by either Δ7-A2 or WT-A2-expressing DCs were found to be indistinguishable with regard to functional avidity, IFN-γ secretion, and degranulation capacity (Fig. S2C). DC-induced *in vitro* expansion of low-frequency, melanocyte/melanoma antigen MART-1 and gp100-specific CD8^+^ T cells from HLA-A2-positive donors was also attempted, but was found to be considerably less robust compared with antiviral T-cell expansion, particularly within the context of a single round of DC stimulation. For these self/tumor antigens, we did not see a statistical difference between DCs expressing Δ7-A2 and DCs expressing WT-A2 at inducing CD8^+^ T-cell expansion from donor PBMC (Fig. S2D).

**Figure 4:**
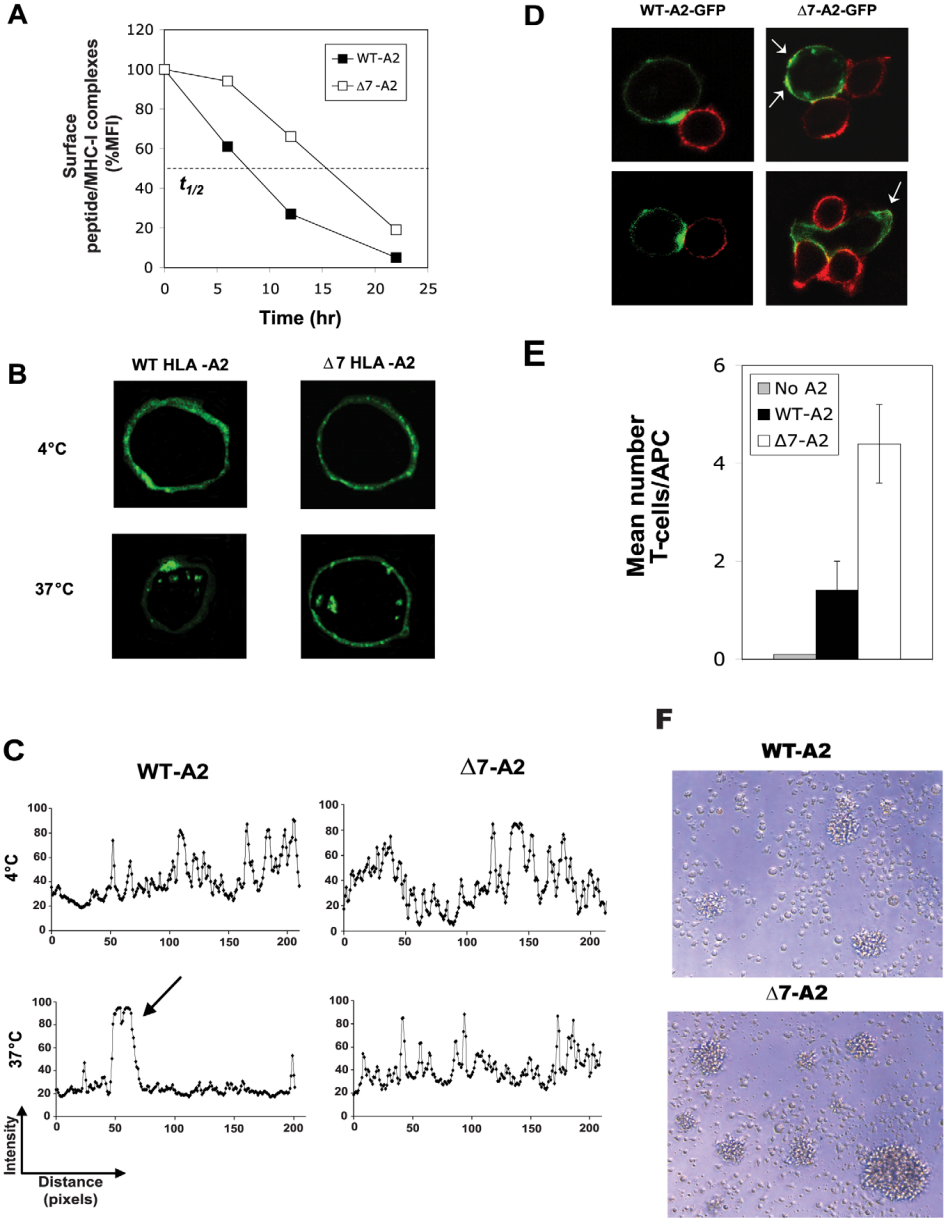
Impaired cell surface mobilization by exon 7-deleted HLA-A*0201 increases bioavailability of MHC-I/peptide complexes for CD8^+^ T cells. (**A**) KG-1 cells transduced to express either WT- or Δ7-HLA-A*0201 were pulsed with MART-1 peptide (20 µM), washed, then stained with a TCR-like mAb specific for HLA-A2/MART-1 complexes at different time points following cell culture at 37°C. Graph depicts the mean fluorescence intensity (MFI) of stained cells, as analyzed by flow cytometry. (**B**) Transduced KG-1 cells were stained with a fluorescently-labeled HLA-A2-specific mAb (green), incubated for 30 min at 4°C or 37°C, and analyzed by confocal microscopy. (**C**) Confocal images from experiments shown in figure 4B were analyzed using Image J to quantify the distribution of WT-A2 and Δ7-A2 molecules at the plasma membrane. Histograms show the pixel intensity at different points within the plasma membrane of transduced KG-1 cells. Arrow indicates clustering of WT-A2 molecules following incubation at 37°C (**D and E**) KG-1 cells transduced to express WT-A2-GFP or Δ7-A2-GFP fusion proteins were pulsed with MART-1(27L) peptide (100 nM), washed, and incubated with MART-1-specific CD8^+^ T cells. After 2 h, cells were fixed, stained for CD3 (red), and (**D**) analyzed by confocal microscopy. White arrows indicate the enhanced spatial plasma membrane distribution of Δ7-A2/GFP molecules. (**E**) Quantification of the mean number of CD8^+^ T-cells per KG-1 antigen-presenting cell in conjugate. (**F**) Typical higher-order, antigen-dependent cell clusters photographed under simple light microscopy following 6 h of DC-T cell co-culture. All results shown are representative of at least 3 replicate experiments.

To gain insights into the mechanisms by which the Δ7 MHC-I splice variant augments CD8^+^ T-cell stimulation, we first utilized a TCR-like mAb that recognizes MART-1/HLA-A*0201 peptide complexes in order to measure surface complex stability over time. Consistent with murine studies [15], Δ7-A2/MART-1 peptide complexes demonstrated extended cell surface half-lives that were approximately twice that of WT-A2/peptide complexes in KG-1 cells (∼16 h vs. ∼8 h, Fig. 4A). Confocal microscopy of transduced KG-1 cells at 4°C revealed similar steady-state plasma membrane distributions of WT and Δ7 HLA-A*0201. However, when the incubation was performed at 37°C, Δ7-A2 molecules demonstrated substantially impaired lateral membrane movement and polar ‘capping’ compared with WT-A2, instead remaining relatively evenly dispersed at the plasma membrane (Figs. 4B and 4C). Furthermore, MART-1-specific CD8^+^ T cells (red) co-cultured with peptide-pulsed KG-1 cells expressing WT-A2-GFP fusion proteins induced very similar clustering of WT-A2-GFP molecules that was invariably localized at the region of the DC membrane in direct contact with the T-cell (Fig. 4D). However, for Δ7-A2-GFP molecules, little or no such membrane clustering was observed in response to CD8^+^ T-cell contact (Fig. 4D). As expected, the increased spatial distribution of membrane Δ7-A2/peptide complexes was also associated with an increase in the mean number of MART-1-specific T cells capable of forming conjugates with co-cultured Δ7-A2 KG-1 cells on a per-APC basis after 2 h of co-culture (Fig. 4E). These results are consistent with those obtained in murine systems (Fig. 1B and [14]). At later time points of co-culture (>6 h), larger order clusters of DC-T cell conjugates formed, typically containing 50 to 300 cells each, which were dependent on the presence of MART-1 peptide (Fig. 4F). APC-T cell clusters containing Δ7-A2-expressing DCs were significantly larger and more numerous than those containing WT-A2 DCs, a difference that was most striking at limiting doses of peptide (Fig. S2E).

## Discussion

These results provide proof of principle that human DC-mediated antigen presentation and subsequent CTL priming responses can be modulated by altering specific elements of the MHC-I cytoplasmic tail. Exon 6-encoded determinants (Tyr-320 in particular) have been shown to be crucial for DC-mediated CTL priming [14], and therefore can be considered a domain that positively regulates CTL responses. Exon 7-encoding determinants, by contrast, appear to *negatively* regulate CTL responses, as evidenced by the superior CD8^+^ T-cell stimulation capacity of Δ7 MHC-I molecules. Sequence analysis of >10,000 human expressed sequence tags (ESTs, http://www.ncbi.nlm.nih.gov/dbEST/) for HLA-A, -B, and –C suggests that exon 7-deleted splice variants of MHC-I do not exist in humans. Although Δ7-MHC-I isoforms have been reported to occur in mice, cows, and chickens, they have not been reported to occur naturally in any primates, perhaps implying that exon 7 splicing may have been lost during evolution. Nonetheless, a Δ7 isoform of HLA-A*0201 showed a significantly enhanced capacity to stimulate human CD8^+^ T cells, suggesting that a potential loss of exon 7 splicing in humans during evolution may have had functional implications for adaptive immunity, potentially providing protection from CTL-mediated autoimmunity or excessive inflammatory responses.

Collectively, our data in both murine and human systems demonstrate that Δ7 MHC-I provides significant advantages over WT molecules for inducing superior CTL responses. Although reduced MHC-I DC surface internalization contributed to this effect, much of the increased stimulatory capacity by Δ7 was observed at early time points of DC-T cell contact (1 to 3 hours), when WT MHC-I molecules had yet to undergo significant internalization. Confocal microscopy studies revealed that CD8^+^ T-cell recognition of cognate antigen on peptide-pulsed APCs induced rapid clustering or ‘capping’ of WT MHC-I molecules at the site of T-cell contact that greatly limited the bio-availability of MHC-I/peptide complexes for CD8^+^ T cells. By contrast, exon 7-deleted MHC-I molecules showed greatly impaired T-cell induced clustering, resulting in increased MHC-I/peptide complex bio-availability and enabling APCs to stimulate more CD8+ T cells on a per-cell basis. As predicted, DCs engineered to express Δ7 MHC-I augmented T-cell mediated anti-tumor immunity and significantly extended mouse survival, suggesting that similar strategies could improve efficacy of human DC vaccines designed to elicit viral or tumor antigen-specific CTL responses. However, looking beyond the engineered expression of specific Δ7 HLA alleles in DCs, it may be more effective to target exon 7-encoded determinants pharmacologically in order to neutralize their negative impact on CTL priming, an approach that could simultaneously improve antigen presentation by all endogenously-expressed HLA-A, -B, and -C alleles.

Many of the cellular mechanisms that govern MHC-I/peptide complex clustering, internalization and turnover in DCs remain to be elucidated. However, exon 7 does encode a highly conserved serine phosphorylation site, Ser-335, that may serve to link MHC-I trafficking and antigen presentation with as-yet unidentified cellular kinases and phosphatases [18], [28]. In addition, exon 6 contains at least two other potential sites for post-translational modifications: a putative phosphorylation site at Tyr-320 [29], and a highly-conserved ubiquitination site at Lys-316 [30]. If these modifications modulate MHC-I function *in vivo* as might be expected, inhibitors of the kinases, phosphatases, or ubiquitin ligases that utilize the MHC-I tail as a substrate could be highly immunomodulatory. Lastly, it will be of interest to determine the nature of cytoskeletal components and trafficking machinery in DCs that bind to or associate with the MHC-I tail, potentially in response to one or more of these modifications. These studies open a door toward the exciting possibility of direct pharmacological manipulation of CTL priming responses at the level of antigen presentation, through direct targeting of the MHC-I cytoplasmic tail.

## Materials and Methods

### Dendritic cell transductions

Bone marrow-derived murine DCs were transduced on culture day 3 with lentiviral vectors encoding WT-D^b^-IRES-GFP, Δ7-D^b^-IRES-GFP, or empty-IRES-GFP at a multiplicity of infection (MOI) of 5 in RPMI, with GM-CSF (50 ng/ml), and polybrene (4 µg/ml) by spinfection, as described (see *Methods S1*). Transduced cells were then cultured for 7 additional days, supplemented with GM-CSF. Cells were collected and analyzed by FacScan (BD Biosciences) for viability and expression of GFP and H-2D^b^ before use. Human HLA-A*0201 negative KG-1 cells [27] or CD34-derived DCs were transduced with lentiviral vectors encoding either WT-HLA-A*0201 or Δ7-HLA-A*0201 in XVivo-15 serum free media (Cambrex) at MOI 5 in polybrene. Cells were then expanded in the appropriate cell media and cultured for 5 days before use. KG-1 cells expressing similar cell surface levels of WT-A2 and Δ7-A2 were isolated by cell sorting. Transduced primary DCs were normalized to ensure that the same number and percentage of WT- and Δ7-MHC-I expressing cells were used for all functional experiments.

### T-cell assays

#### Cytokine secretion

Transduced murine DCs were pulsed with titrated concentrations of hgp100(25–33) peptide, which is a human analog of the mouse gp100(25–33) epitope that is restricted to H-2Db, and co-cultured with effector Pmel-1 T cells at a 1∶10 ratio at 37°C for 18 hrs. To generate effector Pmel-CD8+ T cells (>98% gp100-specific), fresh Pmel spenocytes were cultured for 7 days with 1 µM hgp100 peptide as previously described [6]. For human studies, transduced KG-1 or CD34-derived DCs were pulsed with titrated MART-1(26–35,27L) or FluM1 (58–66) peptides and co-cultured with MART-1 or Flu-specific T cells at a 1∶10 ratio for 18 h at 37°C. Supernatants were collected and cytokine production was measured by ELISA (Endogen) and Luminex (Millipore). For intracellular cytokine staining, T cells were incubated with DCs for 4 h in the presence of GolgiStop (BD Biosciences), washed, fixed, permeabilized, and stained using anti-mouse or anti-human IFN-γ conjugated to FITC (BD Biosciences). Human T cells were also stained with either MART-1 or FluM1 tetramers (Baylor College of Medicine) and a fluorescently labeled anti-human CD8 antibody (BD Biosciences). Antigen-specific intracellular IFN-γ production by CD8^+^ T cells was then determined by flow cytometric analysis.

#### In vitro proliferation

Fresh Pmel splenocytes (naïve Pmel-1 T cells) were labeled with CFSE (Sigma) and co-incubated with D^b^-transduced DCs pulsed with titrated amounts of hgp100 peptide (0.1, 1 and 10 nM) at 37°C for 48, 72 and 96 hours. Following stimulation, cells were harvested and stained using an anti-Thy.1.1 antibody conjugated to APC (eBioscience) to label Pmel T cells. To remove cell debris, PI was also added to the cells prior to flow cytometry analysis as recommended (BD Biosciences). The number of Thy.1.1 Pmel-1 T cells undergoing proliferation was assessed by dilution of CFSE over time. At least 10,000 cells were analyzed in each sample.

HLA-A2-positive peripheral blood mononuclear cells (PBMCs) derived from normal donors were obtained from Gulf Coast Blood Center. Human DCs were pulsed with titrated amounts of peptide in serum-free media for 2 hr at room temperature. HLA-A*0201-restricted peptides used were MART-1 (26–35,27L: ELAGIGILTV), influenza matrix 1 protein (58–66: GILGFVFTL), or CMVpp65 (495–503: NLVPMVATV, Beckman Coulter). Following peptide pulsing, DCs were irradiated with 20,000 rads, washed, and co-cultured with T cells at a ratio of 1∶10 for 8 days. Human recombinant IL-2 (Proleukin) was added to the culture every 2–3 days. Following multiple rounds of stimulation, lines were analyzed by flow cytometry to determine the number of tetramer positive cells in the CD8^+^ T-cell population. We also measured the function of the expanded antigen-specific T cells by measuring intracellular IFN-γ staining and CD107a in response to antigen presentation by T2 cells.

### Adoptive transfer, Vaccination and Tumor treatment

DCs derived from DBA/2 mice were transduced with lentivirus encoding H-2D^b^ variants. C57BL/6 mice (8–10 weeks old) were inoculated subcutaneously with 5×10^5^ B16 melanoma cells. On day 6 or 7 after tumor injection, tumor-bearing mice were subjected to a sublethal dose of irradiation (350 rad). One day after irradiation, pmel-1 T cells (5×10^6^/mouse) pre-mixed with transduced DCs (1×10^6^/mouse) pulsed with 300 nM hgp100_25–33_ peptide were adoptively transferred into the mice. Recombinant human IL-2 (6×10^5^ units twice daily) was administered intraperitoneally for 3 consecutive days. B16 tumor growth was monitored by measuring the perpendicular diameters of tumors. On day 7 and 21 after DC vaccination, the experimental mice were bled to evaluate the percentages of peripheral Thy1.1+ Pmel-1 cells by flow cytometry. Tumors were measured with calipers, and the products of perpendicular diameters were recorded. Animal experiments were approved by the institutional IACUC, and mice were sacrificed when tumors exceeded 15 mm in diameter or became ulcerated or mice became moribund. Experiments were carried out in a blinded, randomized fashion and performed three times with similar results.

## Supporting Information

Figure S1 (**A**) *Top*, Lentiviral gene transfer vectors used to transduce murine DCs. The mouse stem cell virus (MSCV) promoter was used to drive the expression of WT and Δ7 isoforms of H-2D^b^. A downstream internal ribosome entry site (IRES) allowed for the concurrent expression of green fluorescent protein (GFP). *Bottom*, Predicted amino acid sequences of the cytoplasmic domains of WT and Δ7 H-2D^b^. Exon 7-encoding amino acids are depicted as red, and bold font indicates reported phosphorylation sites. (**B**) WT-D^b^/GFP-, Δ7-D^b^/GFP-, or GFP-tranduced DCs were pulsed with titrated concentrations of hgp100(25–33) peptide, washed, and used to stimulate naïve Pmel-1 CD8^+^ T cells. Graph indicates the total number of Pmel-1 T cells present at d4 of co-culture, as quantitated by trypan blue staining. (**C**) Transduced DCs were pulsed with hgp100 peptide (300 nM), and co-injected along with Pmel-1 CD8^+^ T cells into C57BL/6 recipients. DC-mediated T-cell expansion *in vivo* was compared by measuring the percentage of Thy1.1^+^ Pmel-1 T cells in peripheral blood at different time points following adoptive transfer.
(EPS)

Figure S2 (**A**) *Top*, Lentiviral gene transfer vectors used to transduce human KG-1 cells and primary DCs. The human phosphoglycerate kinase (hPGK) promoter was used to drive the expression of WT and Δ7 isoforms of HLA-A*0201. *Bottom*, Predicted amino acid sequences of the cytoplasmic domains of WT and Δ7 HLA-A*0201. Exon 7-encoding amino acids are depicted as red, and bold font indicates reported phosphorylation sites. (**B**) Human DC-like KG-1 cells and primary CD34^+^-derived DCs were transduced to express comparable levels of surface HLA-A*0201, as determined by HLA-A2-specific mAb staining and flow cytometry. WT-A2, grey and blue histograms; Δ7-A2, black and green histograms. (**C**) FluM1-specific CD8^+^ T cells were expanded *in vitro* using WT-A2 or Δ7-A2-expressing DCs. Expanded T cells were then tested for effector function by co-incubating for 4 h with T2 cells pulsed with titrated amounts of FluM1 peptide, then stained for intracellular IFN-γ and surface CD107a. (**D**) Expansion of low frequency, MART-1-specific T cells from peripheral donor blood using peptide-pulsed WT-A2 or Δ7-A2-expressing KG-1 cells as stimulator cells. (**E**) Quantitation of the mean number of APC/T-cell clusters per culture well, as counted in a blinded fashion.
(EPS)

Methods S1 (DOC)
