# Supplementary material for: Natural Splice Variant of MHC Class I Cytoplasmic Tail Enhances Dendritic Cell-Induced CD8+ T-Cell Responses and Boosts Anti-Tumor Immunity
Source: PLoS One. 2011 Aug 10;6(8):e22939. doi: 10.1371/journal.pone.0022939 (PMC3157908; doi:10.1371/journal.pone.0022939)
Supplement: Methods S1 — (DOC) [file pone.0022939.s003.doc]

***Supplementary Materials and Methods:***

**Mice and murine melanoma cells:** T cells from Thy1.1^+^ Pmel-1 transgenic mice express a T cell receptor (TCR) restricted to H-2D^b^ molecules presenting the melanoma tumor antigen, gp100 (gp100_25-33_), as described previously (26). The majority of the CD8^+^ T cells from these transgenic mice were VB13^+^ Thy1.1^+^ (>95%) as measured by flow cytometry. DBA/2, C57BL/6 and Thy1.1+ Pmel-1 transgenic mice were maintained in a pathogen-free facility at the MD Anderson Cancer Center. B16 melanoma cells were obtained from the National Cancer Institute tumor repository (Bethesda, MD) and cultured in complete medium consisting of RPMI 1640 supplemented with 10% heat-inactivated fetal bovine serum, 0.03% L-glutamine, 100ug/ml streptomycin, 100ug/ml penicillin, and 50ug/ml gentamicin sulfate (Invitrogen).

**Human cell culture:** The KG-1 DC-like cell line was obtained from ATCC (**CCL-246™**) and cultured in Iscove's Modified Dulbecco's Medium containing 20% FBS, HEPES, Glutamax, and Penicillin and streptomycin. 293METR cells were a kind gift from Brian Rabinovich (MD Anderson Cancer Center). Cells were cultured in DMEM media supplemented with 10% Fetal Bovine Serum, HEPES, Glutamax, Penicillin/Streptomycin and Normocin (all from Invitrogen). The MART-1 specific TIL line DMF5 was kindly provided by Laura A. Johnson from the NIH. These T cells were cultured in RPMI supplemented with 10% FBS, HEPES, Glutamax, Penicilin/Streptomycin. In addition, human recombinant IL-2 (300 IU/ml) was added every other day.

**Generation of cytoplasmic domain mutants of HLA-A2 and H-2D^b^:** Cloning of HLA-A*0201 (A2) was performed using the Gateway system (Invitrogen). Briefly, wild-type HLA-A2 was amplified from pCDNA.3.1 HLA-A2 (NCBI) using the 5’ primer: **GGGGACAAGTTTGTACAAAAAAGCAGGCTTCGAATTC***GCCACC*ATGGCCGTCATGGCGCCCCGA, containing the recombination site attB1 (bold), an EcoRI site (bold underlined), a Kozak sequence (italics) and the initiation codon (underlined) of HLA-A2. In addition, we used the 3’ primer: **GGGGACCACTTTGTACAAGAAAGCTGGGTC**TCA*ATCGAT*CACTTTACAAGCTGTGAG, containing the recombination site attB2 (bold), the stop codon (underlined) and Cla I site (italics). Following amplification and cloning into pDONOR222 vector (Invitrogen), mutations in the cytoplasmic domain of the gene encoding HLA-A2 were introduced using a PCR-based site-directed mutagenesis kit (Stratagene). To generate pDONOR222 Δ7-HLA-A2, we performed PCR on pDONOR222 WT-HLA-A2 using the following mutagenesis primers: 5’ primer CTCTCAGGCTGCAGTGTGAATCGATG, and 3’ primer CATCGATTCACACTGCAGCCTGAGAG. Novel recombinant plasmids were validated by DNA sequencing. Following sequencing analysis, pDONOR222 WT-HLA-A2 and pDONOR222 Δ7-HLA-A2 were cloned into a self-inactivating lentivirus expression vector containing the human PGK promoter via LR reactions (Gateway technology from Invitrogen). Bacterial transformations were then performed to amplify the expression vector followed by generation of lentivirus virions for transductions.

Cloning of H-2D^b^ was also performed as described above. Wild-type H-2D^b^ cDNA (a kind gift from James Gibbs, NIAID) was amplified using the 5’ primer: **ggggacaagtttgtacaaaaaagcaggct**tc*gccacc*atgggggcgatggctccgcg, containing the recombination site attB1 (bold), a Kozak sequence (italics) and the initiation codon (underlined) of H2-D^b^. In addition, we used the 3’ primer: **GGGGACCACTTTGTACAAGAAAGCTGGGT**CTCACGCTTTACAATCTCGG, containing the recombination site attB2 (bold), and the stop codon (underlined). Following PCR, WT-H-2D^b^ was cloned into pDONOR222 via BP reaction as described (Gateway, Invitrogen). pDONOR222 Δ7-H-2D^b^ was generated by PCR-based site-directed mutagenesis using pDONOR222 WT-H-2D^b^ as a template. The following mutagenesis primers were used: 5’ primer gctctggctccagcgtgagacccagc and 3’ primer gctgggtctcacgctggagccagagc. These constructs were then sequenced and cloned into a self-inactivating lentivirus expression vector containing the mouse stem cell virus promoter via LR reactions.

**Generation of different HLA-A2/GFP fusion proteins:** To generate WT-HLA-A2/GFP and Δ7-HLA-A2/GFP fusion proteins, we used pENTR1A containing GFP and a G4S linker downstream of the death cassette (a kind gift from Brian Rabinovich, M.D. Anderson) and a cloning site made of EcoR1 and Cla I sites flanking the death cassette as template. HLa-A2 Wt and HLA-A2 Δ7 were amplified using the primers described above with some modifications. (1) The primers did not have the attB1 and attB2 site and (2) the 3’ primer did not contain the stop codon of HLA-A2. The molecules were then cloned into pENTR1A GFP between the EcoRI and Cla I site by ligations. Constructs were confirmed by DNA sequencing. Following DNA confirmation, plasmids were then cloned into the human PGK self-inactivating lentivirus as mentioned above.

**Lentivirus generation:** Lentivirus virions were generated by transient transfection of the packaging cell line 293METR with plasmids expressing either wild type (WT)-HLA-A2, Δ7-HLA-A2, WT-HLA-A2/GFP, Δ7-HLA-A2/GFP, WT-H-2D^b^ or Δ7-H-2D^b^ (2.3μg each), along with p∆R8.91 (4.7μg) and CMV-pVSVG (2μg) using Lipofectamine 2000 (1.6ug/ml). Viral supernatants were collected at 48 and 72 hrs, subjected to ultracentrifugation at 20,000*g*. Viral pellets were resuspended at a 400X concentrate in X-vivo15 serum-free medium. Lentiviral titers averaged in the 2x10^8^ to 1x10^9^ IU/ml range.

**Lentivirus titration:** Approximately 30,000 293 METR cells per well were cultured in 1ml DMEM to all wells of a 24-well plate and incubated for 18 hrs at 37°C. The following day, media was removed and 0.2 ml of fresh media was added to the cells for titrations. Three-fold serial dilutions of lentiviral vector stock were then added to the cells along with 8 μg/ml of polybrene (Sigma) to enhance viral transduction efficiency. Cells were then trypsinized and collected to measure GFP expression by flow cytometry. Viral vector concentrations were calculated as follows: Transducing units (TU) per ml = % GFP transgene positive cells x 30,000 cells / volume of viral vector used (in ml).

**Generation of DCs:** Bone marrow-derived murine DC progenitors were isolated from the tibia and femur of DBA/2 mice. Cells were cultured in complete medium consisting of RPMI 1640 supplemented with 10% heat-inactivated fetal bovine serum and GM-CSF (50ng/ml) at a starting concentration of 5×10^5^ cells/ml.

CD34^+^ cells were purified from HLA-A2 negative donor stem cells (a kind gift from Nina Shah, M.D. Anderson) using CD34^+^ selection beads (Miltenyi). Purified CD34^+^ cells were then expanded for 2 weeks with a media containing Flt3L (100ng/ml), TPO (100ng/ml) and stem cell factor (100ng/ml). Following expansion, CD34^+^ cells were transduced with lentivirus vectors encoding either WT or Δ7 HLA-A2 in the presence of Flt3L (100ng/ml), TPO (100ng/ml) and Stem cell factor (100ng/ml). Cells were incubated overnight with the lentiviral vectors at 37°C. The next day, cells were washed and incubated for 4 additional days at 37°C in the presence of the cytokines described above. Cells were then washed and incubated at 37°C in the presence of human recombinant GM-CSF and IL-4 for 6 additional days in order to induce the differentiation of immature DCs. To mature DCs, a combination of poly:IC, IFN-alpha and CD40L were added to the culture for 1 additional day.

**Internalization of cell surface HLA-A2 in human dendritic cells.** In order to assess the kinetics of internalization of HLA-A2 molecules, human dendritic cells expressing either HLA-A2 WT or Δ7 were first labeled with an Fc blocking antibody for 30 minutes at 4°C to exclude binding of antibodies to Fc receptors. Cells were then stained on ice for 30 minutes by using a monoclonal fluorescently labeled HLA-A2 specific antibody (Serotec). Cells were washed and incubated at either 37°C or 4°C for 90 minutes. The remaining fluorescence at the cell surface following warming cells to 37°C was measured by confocal microscopy (Leica) as previously described (15). HLA-A2 clustering was quantified using Image J software (<http://rsbweb.nih.gov/ij/>).

**APC/T-cell conjugates and confocal microscopy:** KG-1 cells expressing WT-A2/GFP or Δ7-A2/GFP were pulsed with MART-1 peptide (20nM) for 2 hrs at room temperature. Cells were then washed, mixed with equal amounts of MART-1 specific T cells, plated onto poly-L-lysine-coated coverslips and incubated at 37°C for up to 2hrs. Cells were fixed using 4% PFA (Fisher) and stained with anti-CD3-ζ (6B10.2) mAb conjugated to alexa 647 on ice to label MART-1 T cells. APC/T cell conjugates were visualized on a Leica TCS SP2 confocal microscope using a 60X oil objective. Images were collected as Z-stacks with 20 planes and 2μM spacing between each plane. KG-1-T cell conjugates were quantified by visually counting numbers of T cells conjugated to a random selection of 20 to 30 KG-1 cells. Conjugates were scored only if T cells were in contact with single GFP-labeled KG-1 cells.

**Statistical analysis:** The statistical analyses to compare mouse survival between treatment and control groups were determined with a Kaplan-Meier test using Prism version software. Tumor growth statistics were determined using an unpaired T-test. Unless noted, data are presented as the mean +/- SE of data from five to eight mice per group.
